# The Treatment of Vomiting in Tuberculosis

**Published:** 1897-04

**Authors:** 


### THE TREATMENT OF VOMITING IN TUBERCULOSIS.

In the *Revue de Thérapeutique Médivo-Chirurgical*, of December 1, 1896, Mathieu is reported to have recommended the employment of the following treatment in this troublesome condition. He first points out that the vomiting largely depends upon the fact that the stomach is reflexly irritated from the pneumogastric nerve by the diseased condition of the lung. For this reason small pieces of ice or one to two teaspoonfuls of chloroform water may be given every ten minutes for several days to allay gastric irritability after food is taken. After the meal the following mixture may be given:

℞ Menthol, 3 grains.
Syrup, 5 ounces.

The chloroform, however, constitutes the most efficacious form of medication.

In the discussion which followed Mathieu's paper, Ferrand stated that he had gotten the best results from diminishing the sensibility of the pharynx by the application of 10 per cent. solutions of bromide of potassium in glycerin.—*The Therapeutic Gazette.*

---

Bismuth Paste for Orchitis.—A thick paste (*Southern Practitioner*) consisting of subnitrate of bismuth and water is the best application for swollen testicles. It relieves the pain and the burning sensation, and the swelling rapidly subsides. It is equally successful for burns and as an application for sunburn and blistered skin.

